# Complete genomes of *Salmonella* and draft genome of *Escherichia* isolates from oysters collected in Canada

**DOI:** 10.1128/mra.00585-24

**Published:** 2024-12-13

**Authors:** Bojan Shutinoski, Annika Flint, Sandeep Tamber, Kelly Weedmark, Swapan Banerjee

**Affiliations:** 1 Bureau of Microbial Hazards, Health Canada, Ottawa, Ontario, Canada; University of Maryland School of Medicine, Baltimore, Maryland, USA

**Keywords:** *Salmonella*, *Escherichia coli*

## Abstract

Human enteric pathogens in fresh uncooked seafood are of concern to human health. Here, we report the complete genomes of bacteria not commonly found in freshly harvested seafood, two *Salmonella* Typhimurium strains and one draft *Escherichia coli* genome, isolated from harvested oysters grown in Canada.

## ANNOUNCEMENT

Seafood poisoning due to the consumption of raw or undercooked mollusks is predominantly associated with bacteria belonging to the *Vibrio* genus (1), which are native to the marine environment. Nonetheless, other types of bacteria and pathogens—e.g., viruses and parasites—are relevant to seafood safety (2–5). The present study sequenced the genomes of two *Salmonella enterica* serovar Typhimurium isolates and one *Escherichia coli* isolates from oysters.

Freshly harvested oyster samples collected by the Canadian Food Inspection Agency were processed to isolate single bacterial colonies. For *Salmonella,* 50 g of shucked oysters were homogenized in a blender and incubated overnight at 35°C in 450 mL of Buffered Peptone Water (BPW), pH 7.0. Secondary enrichment was carried out in Rappaport-Vassiliadis soya (RVS) broth by inoculating 100 µL of BPW primary enrichment in 10 mL of RVS at 41.5°C, 150 rpm, 18–20h. RVS enrichment (10 µL) was streaked on Xylose Lysine Deoxycholate agar plates from which single colonies were isolated (6). For *E. coli*, 50 g of shucked oysters were homogenized in a blender and incubated overnight at 35°C in 450 mL of 1% Bacto-peptone, pH 8.5, and 10 µL was streaked on CHROMagar *Vibrio* (Dalynn Biologicals) to isolate single colonies. The −80°C stocks were prepared from a single colony (10 µL loopful/Microbank vial-VWR).

For sequencing, stocks were streaked onto tryptic soy agar with 2% NaCl (TSA-2N) and incubated at 35°C overnight. A single colony was inoculated in 5 mL TSA broth at 35°C overnight, no shaking, and 500 µL of culture was added to an equal volume of 2× Zymo DNA/RNA Shield (Cedarlane). DNA was extracted using the Zymo Quick-DNA HMW Magbead kit (with RNAseA treatment) as per the manufacturer (Zymo Research Corp). Paired-end Illumina sequencing was performed on all isolates using Nextera XT libraries run on a MiSeq instrument (2 × 300 bp cycles, v3 chemistry) according to the manufacturer (Illumina, Inc.). Oxford Nanopore sequencing was performed on the two *Salmonella* isolates using the rapid barcoding sequencing kit (SQK-RBK004) and 1D MinION chemistry (R9.4 FLO-MIN106 flow cell) as per the manufacturer (Oxford Nanopore Technologies).

All computational tools used default settings except where specified. Illumina reads were processed using FastP-v0.20.1 to remove barcode sequences, correct mismatched bases, and filter low-quality reads (Q < 20) (7). Long-read signal processing, base calling, demultiplexing, and adapter trimming were performed using Guppy GPU-v6.0.1+652ffd1 (community.nanoporetech.com), and reads of <5 kb were removed using Filtlong-v0.2.0 (github.com/rrwick/Filtlong). *De novo* assemblies were generated and rotated to the starting gene using Unicycler-v0.5.0 in normal mode (8) and error corrected using Medaka v1.1.3 (github.com/nanoporetech/medaka), Polypolish-v0.4.3 (9), and Polca-v4.0.5 (github.com/alekseyzimin/masurca). Assemblies were annotated with PGAP-v2022-04-14.build6021 (best-placed reference protein set, GeneMarkS-2+) (github.com/ncbi/pgap) and analyzed with QUAST-v5.0.2 (github.com/ablab/quast). Taxonomic assignment was confirmed using FastANI-v0.1.3 on KBase (accessed September 2022) (10).

Sequencing and assembly details are listed in Table 1. Raw coverage depths ranged from of 96- to 445-fold. The two *Salmonella* genomes are closed compromising a 4.9 Mbp circular chromosome and two circular plasmids (94 kb and 2 kb). The *Escherichia* genome is a 4.9 Mbp draft genome with 57 contigs.

**TABLE 1:** Isolate metadata, sequencing metrics, NCBI SRA accession, genome assembly/annotation metrics, and NCBI accession numbers (BioProject PRJNA880531)^*a*^

Isolate	HS-180Salm		HS-187Salm		HS-228-7
Organism	*S*. Typhimurium		*S*. Typhimurium		*E. coli*
Harvest location	Canada, NB		Canada, NB		Canada, NB
Date	2021-Aug-21		2021-Sep-15		2022-Aug-09
Sequencing technology	Illumina	ONT	Illumina	ONT	Illumina
Raw reads (#)	3,072,120	348,931	2,022,636	357,699	1,596,032
Average raw length (N50) (bp)	298	6,182 (11,778)	298	6,103 (11,519)	296
Raw fold coverage	187×	440×	123×	445×	96×
Filtered reads (#)	1,377,318	138,741	520,828	141,040	1,441,304
Average filtered length (N50) (bp)	296	12,499 (14,540)	292	12,380 (14,296)	295
SRA accession no.	SRR21564523	SRR21564521	SRR21564522	SRR21564520	SRR25036427
BioSample		SAMN30847003		SAMN30847004	SAMN35996173
Total genome size (N50) (bp)		4,894,501		4,894,979	4,932,197 (294,736)
# Contigs		3		3	57
GenBank accession no.		CP129209		CP129206	JAUEQZ000000000
		(4.9 Mb Chromosome)		(4.9 Mb Chromosome)	
		CP129210		CP129207	
		(94 kb plasmid01)		(94 kb plasmid01)	
		CP129211		CP129208	
		(1.9 kb plasmid02)		(1.9 kb plasmid02)	
GC content (%)		52.19		52.19	50.61
Total features		4,871		4,873	4,821
Genes		4,592		4,592	4,593
rRNAs		22		22	1
tRNAs		85		85	77
ncRNAs		16		15	9
Pseudogenes		156		159	141
CRISPRS		3		3	4

^*a*^ NB, New Brunswick; ONT, for Oxford Nanopore Technologies.

## Data Availability

The genome assemblies for HS-180Salm, HS-187Salm, and HS-228-7 have been deposited in GenBank under accession numbers CP129209-10-11, CP129206-07-08, and JAUEQZ000000000, respectively. All SRAs are available in GenBank under BioProject ID PRJNA880531.
